# A Community-based Bacteriological Study of Quality of Drinking-water and Its Feedback to a Rural Community in Western Maharashtra, India

**Published:** 2008-06

**Authors:** Prachi V. Tambe, Poonam G. Daswani, Nerges F. Mistry, Appasaheb A. Ghadge, Noshir H. Antia

**Affiliations:** ^1^Foundation for Medical Research, 84-A, R.G. Thadani Marg, Worli, Mumbai 400 018, Maharashtra, India; ^2^Foundation for Research in Community Health, 3 & 4, Trimiti-B Apartments, 85, Anand Park, Aundh, Pune 411 007, Maharashtra, India

**Keywords:** Community participation, Diarrhoea, Drinking-water, Interventions, Longitudinal studies, Water pollution, Water supply, India

## Abstract

A longitudinal study of the bacteriological quality of rural water supplies was undertaken for a movement towards self-help against diseases, such as diarrhoea, and improved water management through increased community participation. Three hundred and thirteen water samples from different sources, such as well, tank, community standpost, handpumps, percolation lakes, and streams, and from households were collected from six villages in Maharashtra, India, over a one-year period. Overall, 49.8% of the 313 samples were polluted, whereas 45.9% of the samples from piped water supply were polluted. The quali-ty of groundwater was generally good compared to open wells. Irregular and/or inadequate treatment of water, lack of drainage systems, and domestic washing near the wells led to deterioration in the quality of water. No major diarrhoeal epidemics were recorded during the study, although a few sporadic cases were noted during the rainy season. As a result of a continuous feedback of bacteriological findings to the community, perceptions of the people changed with time. An increased awareness was observed through active participation of the people cutting across age-groups and different socioeconomic strata of the society in village activities.

## INTRODUCTION

It is well-appreciated that many communities in developing countries face severe public-health problems relating to drinking-water (1). The supply of safe water is important to protect the health of the community people (2). Scarcity and pollution of water—both microbial and chemical—are major problems faced by rural population in several parts of India (3,4). Lack of sanitation is detrimental to water potability concentrating pathogenic organisms. Diarrhoea is the most common infectious disease worldwide (5), and gastrointestinal infections kill 1.8 million people globally each year, mostly children in developing countries (6). While urban industrial centres in such areas continue to receive water supplies from rural lakes and water sources by virtue of their economic status, rural communities with no traditional base of harvesting and conservation of water are left in the lap of migration and/or destitution.

The Parinche valley in Purandar block of interior Western Maharashtra, India, is one such area where the above conditions prevail. Average annual rainfall in this area has receded by approximately 50% in the last four years.

The approach in this study was grounded in the belief that amelioration of this situation will ultimately rest with the efforts of the local community. Given the iniquitous water distribution and lack of community control of this vital natural resource, understanding of issues relating to both quality and quantity of water becomes necessary (7). One way of generating community focus on issues concerning drinking-water comprises generating and sharing of bacteriological information at multiple points of supply/distribution, storage, and use. The concurrent linkage of sporadic epidemics of diarrhoea occurring in an area to quality and availabili-ty of water changes the perceptions of the people about the risk of diarrhoeal disease (8). Thus, a feedback on bacteriological quality of water to the community was an intrinsic part of this study for engendering a two-way partnership between external outputs and internal processes of the community. Transfer of technological knowledge and its social advocacy were the expected outputs.

A catalytic presence of female health and development workers in the valley, trained by Foundation for Research in Community Health (FRCH), Pune, in preventive, promotive and curative health, facilitated the above-mentioned approach (9). Their presence permitted a close follow-up and observation of participating households. Their ability to network and provide information to communities and their active participation in local governance fostered discussion, advocacy, and implementation of learnings gained from technological inputs (10).

Delving into bacteriology was judged to have a better effect if the observations were made closer to each home. Hence, a longitudinal survey was planned from the wells and tanks to the common tap (referred as standpost) and eventually the stored household supplies. Regular water-supply cycles in all villages, including source of water, duration and frequency of supply, and seasonal changes in source/frequency, were studied along with the water-storage practices at the household level. The study began with the objectives of assessing the bacteriological quality of water sources and maintaining constant discussion with the community and its facilitators, evolving joint solutions, and creating awareness. We also aimed at effecting changes in the perceptions and water-storing practices by the community. Ultimately, to disinfect water at the point-of-use in the households, a novel technological intervention in the form of coiled copper wire was introduced (11). Its inherent advantages are described subsequently.

## MATERIALS AND METHODS

### Baseline information

### Rural project area

Situated 60 km south of Pune, the project area was divided into three major valleys of Parinche, Pangare, and Kaldari designated for ease as Area A, B, and C respectively.

### About Foundation for Research in Community Health

Established in 1975 as a non-profit voluntary organization, the FRCH carries out field studies and conceptual studies primarily in rural India. At the time of this study, the FRCH has been functioning in the study area for over seven years. Its thrust during this time was to develop and demonstrate the functional scope of a cadre of female community health workers (CHWs) for a community-based healthcare system (9).

### Role of CHWs

The CHWs, selected through a participative process of community consensus, were trained over a one-year period in preventive, promotive and curative aspects of health and medical care. Operating at a favourable ratio of one worker for 20–25 households, the CHW provided equitable and high accessibility to primary healthcare to their neighbourhoods marked by intimate contact. This served the requirement of this study for detection and recording cases of diarrhoea, household-water storage/handling practices, and use of proposed technical interventions and link between those who undertook the bacteriological testing of water and the community.

### Concurrent strategies for engendering a two-way partnership

The trained field workers and CHWs from the FRCH gathered detailed information through meetings with the community and the local governing bodies (*Gram Panchayats*). Various modes of community interactions included regular meetings, formation of focus-discussion groups, visits to households, close observations by the CHW, regular communications with and interviewing families, bimonthly poster newsletter called ‘*Maitri*' (friendship), and regular notice-board writing covering various health issues. Occasionally, street plays were organized. An ‘Eco (ecological) group' comprising village school children was formed. They were trained in determination of water potability, measurement of residual chlorine in water sources, conservation of water, and propagation and use of local medicinal plants.

These strategies yielded important information about the sources of water, its supply, including duration/frequency, treatment, practices of storage/handling water within the community, and possible sources of contamination. Observations with respect to sanitary issues included absence of toilets, open wells, manual handling of water from wells, proximity of wells to streams and domestic animals, unclean tanks with occasional leakages, poor drainage, and washing activities near the sources.

The intimate relationship of field workers and CHWs with the community also enabled recording of the acceptance of proposed interventions for improving/maintaining the quality of water at the household level. These included use of coiled copper wire/vessel, use of a chlorine solution (50 g of TCL powder in one litre of water, commonly referred to as ‘mother solution' probably since chlorination of water is thought to be the responsibility of the women), fitting of tap to water-storage vessel, and use of ladle to draw water from the storage vessel. In addition, qualitative data indicating community attitudinal changes could be recorded through close and sustained observations.

### Project area

*Details of villages* (Table 1): Two villages from each area, namely Parinche and Shindewadi (Area A), New Hargude and Kambalwadi (Area B), and Kharadwadi and Kondkewadi (Area C), were selected on the basis of presence of CHWs and variability in terms of the number of common and private water taps, water-treatment practices, and frequency of water supply. Two (Parinche and Konkewadi) of these villages had experienced epidemics of diarrhoea.

**Table 1:** Details of villages selected for the study

Parameter	Village					
	Parinche (Area A)		Pangare (Area B)		Kaldari (Area C)	
	Parinche	Shindewadi	New Hargude	Kambalwadi	Kharadwadi	Kondkewadi
Accessibility	Easy	Difficult	Easy	Difficult	Easy	Difficult
Population	2,000	175	264	264	112	250
Main occupation	Agriculture, milk vending	Agriculture	Agriculture	Agriculture	Agriculture	Agriculture
Water tank capacity (litres)	100,000	15,000	10,000	14,400	5,600	3,000
Sources of water	Well, standpost, handpump	Well, standpost	Well, standpost, handpump	Well, standpost	Well, standpost, percolation lake	Well, standpost, stream
No. of common/private taps	Nil/284	4/7	3/11	4/Nil	3/Nil	3/Nil
Water treatment	Regular, adequate	Irregular	Irregular	Regular, adequate	Only in rainy season	Irregular, inadequate
Water supply	Twice a day	Once a day	Once a day	Once a day	Once a day	Once a day
Frequency duration	Half an hour (once in summer)	Half an hour	Half an hour	Half an hour	Half an hour	Half an hour
Recent diarrhoeal epidemic	Yes (1998)	No	No		No	Yes (1997)

*Water-related practices:* Regular cycle of water supply in the villages comprised water being pumped into the tank from a well and further supply through pipelines. The study area being situated on hard rock, the depth of wells in these villages could not be increased beyond 9–12 metres. It would be an expensive and fruitless exercise to attempt collecting water below this depth. Hence, arrangements, such as supplying water from a nearby dam or percolation through bundings and percolation tanks, were attempted to increase the level of well-water. Chlorination was irregular and/or inadequate with regular electricity failures. The drainage systems were poor. Watermen did not seem to perform satisfactorily. They complained about low payments, efforts needed for treating tanks at a higher level, irregular supply of TCL, etc. People were hesitant to take any initiative in the maintenance of water sources and proper distribution.

*Details of households* (Table 2): Eighteen families (three each from the six villages), using the same water source (tap), were chosen. Table 2 shows that a large majority comprised open caste category (15/18), had middle-class economic status (15/18), and had family heads who completed their secondary school education (16/18). Agriculture was the main occupation. While the socioeconomic and education status was largely similar in most households (exceptions being from Parinche and Kondkewadi), the most significant difference was in the water-storing practices as discussed below.

**Table 2:** Details of households for three villages short-listed for the study

Area	Village	Household	Category	Economic status	Education	No. of children	Occupation	Water-storage level	Type of vessel used
A	Parinche	1	Open	Middle class	Middle school	2	Farmer	Low	Steel, brass
		2	Open	Well to do	Postgraduate	0	Teacher	Low	Steel
		3	Open	Below poverty-line	Middle school	3	Laborer	Low	Steel
	Shindewadi	4	Open	Middle class	Middle school	1	Farmer	High	Steel, brass
		5	Open	Middle class	Middle school	4	Farmer	High	Steel, brass
		6	Open	Middle class	Middle school	2	Farmer	High	Steel, brass
B	Kambalwadi	7	Open	Middle class	Middle school	2	Farmer	Low	Steel, brass
		8	Open	Middle class	Middle school	7	Farmer	High	Steel, brass
		9	Open	Middle class	Middle school	5	Farmer	Low	Steel, brass
	New Hargude	10	Open	Middle class	Middle school	3	Service	Low	Steel, brass
		11	Open	Middle class	Middle school	2	Farmer	Low	Steel, brass
		12	Open	Middle class	Middle school	3	Service	High	Steel, brass
C	Kharadwadi	15	Open	Middle class	Middle school	0	Farmer	High	Steel, brass, copper
		14	Open	Middle class	Middle school	1	Farmer	High	Steel, brass, copper
		15	Open	Middle class	Middle school	2	Farmer	High	Steel, brass, copper
	Kondkewadi	16	Scheduled Tribe	Middle class	Middle school	3	Teacher	Low	Steel, brass
		17	Scheduled Tribe	Middle class	Illiterate	2	Laborer	Low	Steel, brass
		18	Scheduled Tribe	Below poverty-line	Illiterate	0	Laborer	Low	Steel, brass

*Water-related practices:* People stored drinking-water largely in stainless steel or brass vessels and seldom kept covered. The water-pots were usually kept at lower levels. A few followed the traditional practice of washing hands after returning home. However, withdrawal of drinking-water without a ladle was noted frequently. Water was treated by filtration/boiling, only if it looked turbid/muddy or during the monsoon. Most families kept animals near or inside the houses as few could afford separate sheds for animals.

### Testing of water samples

#### Collection and transport of samples

Sampling was done during September 2000–September 2001. Field workers of the FRCH were trained at the Foundation for Medical Research (FMR), a reputable research organization in Mumbai, to collect water using the standard protocol (12). Although an attempt was made to study seasonal variations, this point has not been stressed in this paper as no obvious differences were noted. During the first three months of random testing, a few samples were found to be polluted. It was, therefore, decided to test the samples from all the sources, i.e. wells, tanks, and taps, simultaneously to detect at which level pollution was occurring, and all selected households used the selected standposts. Additionally, it was thought to be necessary to test alternate sources that were used in the case of scarcity of water. Initially, sampling was done once every season (three months) and later twice.

Testing was performed at the FMR approximately 220 km away from the point of collection. Samples were collected in 500-ml sterile plastic bottles containing 18 mg/L sodium thiosulphate solution for treated samples (12) and transported with freezer packs and dry ice within 24 hours to the FMR (if delayed, samples were not tested), and temperature on arrival was 10±2 °C. In pilot experiments, dummy runs with spiked water samples were undertaken to ascertain that no significant drop in viability ensued during transport. Information, such as treatment of water, conditions of areas surrounding standposts, and the drainage system, was noted. Samples were tested for residual chlorine with a commercial chlorine-testing kit, and its status (treated/untreated) was determined.

#### Number of samples tested

*Main sources:* Thirty-two well-water, 17 tank-water, 19 standpost and 99 household samples were tested.

*Alternate sources:* These were categorized into two groups based on their usage; group 1: drinking water during water shortage and group 2: water used for agriculture, domestic washing, and animals. Group 1 included four handpumps from Area A and B and four wells from Area A and C. Group 2 included seven wells—three each in Area A and B and one in Area C, along with a percolation lake and a natural stream from Area C. None of these sources was treated.

Twenty-one handpump samples (Group 1) and 72 Group 2 samples were tested.

#### Bacteriological testing

Samples were tested for coliforms by the Most Probable Number technique (12). As per the Bureau of Indian Standards (13), samples with 0 coliform/100 mL of original water are to be considered excellent, with 1–10 coliform(s) as acceptable, and above 10 coliforms as polluted. For simplicity, samples with 0–10 coliform(s)/100 mL of original water were considered to be acceptable and above 10 coliforms as polluted.

The term ‘clean' has been used throughout for water taken as acceptable. Thus, only the terms ‘clean' and ‘polluted' have been used for indicating the quality of water.

#### Laboratory testing of bactericidal activity of coiled copper wire

1×10^3^/mL log phase culture of *Escherichia coli*, isolated from one of the water samples tested, representative of grossly-polluted water (13), was inoculated in 1 L of sterile phosphate buffered saline (PBS) alone (control) and PBS containing sterilized coiled copper wire (test) of one inch, 8 cm^2^ surface area, procured from a local electric shop. Following incubation at 37^o^C for 3–20 hours, the number of colony-forming units (cfus) in the control and test was determined by plating on nutrient agar. This was repeated thrice, and the results were analyzed by paired *t*-test.

## RESULTS

### Bacteriology

#### Main sources (Fig. 1)

**Fig. 1:**
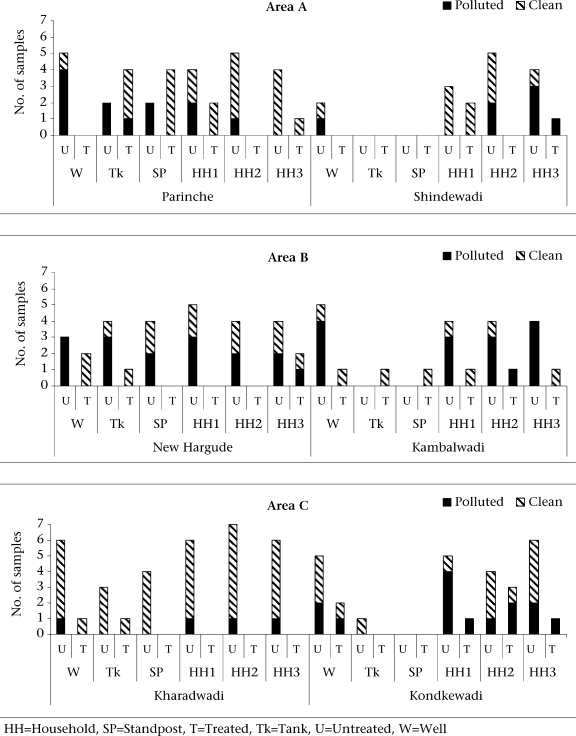
Quality of water in three areas

*Wells:* Thirty-two well-water samples—26 being untreated and six treated—were studied. Five treated samples were clean, and only one was polluted. Whereas, nine untreated samples were clean, and 15 were polluted. The probable reason behind nine clean but untreated samples could be that, while the residual chlorine treatment of the previous day could not be detected, its sanitizing effect was manifested.

*Tanks:* Seventeen tank-water samples—10 being untreated and seven treated—were studied. Five untreated samples were clean, and the remaining samples were polluted. Whereas, six treated samples were clean, and only one was polluted.

*Standposts:* Fifteen of the 19 standpost samples tested were clean. Moreover, samples from 15 of these standposts were compared with those of their respective main sources. All five samples from standposts with treated sources were clean. Additionally, samples from six of the remaining 10 standposts with untreated main sources were also clean. It was noted that, whenever clean tank water was supplied, the tap water was also clean (10/18), thereby indicating that, overall, the engineering aspect of laying of pipelines from tanks to standpost was satisfactory.

*Households:* Ninety-nine household samples were studied. Sixteen samples were treated with either the mother solution or copper wire. However, only nine samples were clean. Of the remaining 84 untreated samples, 49 were clean.

The quality of household water samples in relation to the source is comprehensively depicted in Table 3. Overall, the water-supply pipelines appeared to be in good condition, since a significant number of household sources have a clean water supply. Despite the source being polluted, the households in Area A appeared to have the largest proportion of clean household water (observation 5), Area C being the worst, followed closely by Area B (observation 4). The recontamination at the household level was minimal in Area C (observation 1). The predominantly poor quality of samples at source level (observation 2) highlighted the default of watermen hired by the *Panchayats* to adequately chlorinate the sources.

**Table 3:** Quality of household water samples in relation to the source

Source of water	Area		
	A	B	C*
	(n=19)	(n=17)	(n=17)
Observations			
Tank and standpost clean; household clean	8	8	14
Tank and standpost polluted; household polluted	5	6	0
Tank clean and standpost polluted; household polluted	0	0	0
Tank and standpost clean; household polluted	0	2	3
Tank and standpost polluted; household clean	6	1	0

*Area C represents only Kharadwadi as tank water could not be collected from Kondkewadi

#### Alternate sources

*Group 1:* All 21 handpump samples were found to be clean. Of the eight household samples collected from handpumps, seven were clean. Samples from four wells—three from Area B and one from area C—were untreated and found to be polluted. Household samples from Area B that used water from these wells were also polluted.

*Group 2:* Of the 72 samples collected from group 2 alternate sources, 22 were clean (Table 4).

**Table 4:** Quality of group 2 alternate sources each sampled eight times during the study period

Quality of water	Village										
	Parinche	Shindewadi		New Hargude			Kambalwadi		Kharadwadi	Kondkewadi	
	Area										
	A		A		B	B			C		C
	W1	W2	W		W	W1		W2	PL	W	S
Clean	0	1	1		4	5		4	1	4	2
Polluted	8	7	7		4	3		4	7	4	6

PL=Percolation lake; S=Stream; W=Well

### Bactericidal activity of coiled copper wire

**Fig. 2:**
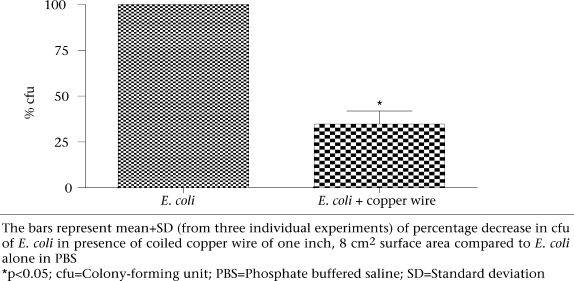
Antibacterial activity of copper wire against *E. coli*

There was a significant reduction in growth of *E. coli* in PBS in which the wire was inserted. This bactericidal activity maximal at eight hours (Fig. 2) remained unchanged on subsequent incubation.

### Acceptance of technical interventions by the community

Table 5 outlines the acceptance of various proposed interventions advocated to the community by the CHW to improve the bacteriological quality of drinking-water. Discontinuation of fitting of taps in Area C (§, Table 5) was largely a result of lack of human resources to replace/repair leaking taps and the expense involved. Surprisingly, the use of ladles had the least takes.

**Table 5:** Acceptance of interventions in families from three areas during and at the end of the study

Intervention	Area A				Area B				Area C			
	Parinche (n=414*)		Shindewadi (n=43*)		New Hargude (n=43*)		Kambalwadi (n=36*)		Kharadwadi (n=30*)		Konkewadi (n=40*)	
	During	After	During	After	During	After	During	After	During	After	During	After
Use of copper coil/vessel	24	190	0	11	3	6	2	5	3	27	2	7
Addition of mother solution	42	140	5	25	5	3	4	35	3	2	4	5
Tap fitted onto vessel	80	85	7	15	8	11	4	35	10	6^§^	8	13
Use of ladle to draw water	5	86	0	0	0	0	0	0	0	0	0	2

*Number of households;

^§^Refer to Results section

### Collective actions taken by the community

At the onset of the study, a passive attitude of the community was observed towards the available supply of water in the village and ignorance about its quality. Through various concurrent strategies outlined earlier, a positive action of the community was observed towards tackling the problem of availability of water. The Box summarizes the collective action taken by the locals.

## DISCUSSION

The bacteriological testing of water quality in this study is not to be considered a technological intervention but rather a facilitating approach supplemented by health education, interactions with local governance, and more effective networking within the community. This facilitating approach, along with concurrent strategies, outlined in the metho-dology section, generated an increased awareness among the locals. As the Box depicts, attitudinal changes viewing water as a community rather than a private resource were apparent. A rights-based approach to availability of water was also discerned. Training of Eco-group in testing of water potability was particularly powerful for questioning of potability results reported by public-health laboratories which were often discordant with the own testing and observations of communities provided by the referral laboratories in Mumbai (data not shown).

The factors for sustenance of interest were identified to be local school children and CHWs who sensitized not only their own families but also the entire community and made use of technical knowledge for persuasion of better measures of governance. The differences in educational and socioeconomic levels in the study areas also appeared to be significant for acceptance of the messages. Area A with a relatively-affluent community and articulate health functionaries were the first to contribute money and effort for solutions, such as a new pipeline.

In the present study, water samples from all possible sources (main/alternate) were tested. According to Wright *et al.*, if water testing is performed only at source, the outcome of testing may not reflect the quality of water actually consumed at home (14). Therefore, water from households was also tested. As the chosen households used the same tap, the results of water testing from these reflect their water-storage/handling practices.

The results indicated that most well-water samples were not treated and, therefore, polluted. Identification of handpumps as safer sources diverted people towards their use more than open wells. The samples from the tanks were polluted in all the villages, except Parinche where regular treatment was observed. A regular cleaning of the tank in Kharadwadi resulted in consistently-acceptable quality of water. Probably due to the past incidence of diarrhoeal epidemic in Parinche (Table 1), tank water was regularly chlorinated. Good quality of water from standposts in Kharadwadi could be due to acceptable quality of tank water. However, observations in Kondkewadi, another village from the same area, are difficult to explain.

In households, the main problem was availability of water, followed by casual handling of water especially by children. This appeared to be the prime cause of reinfection at the household level. Table 3 shows that, in all the three areas, a large proportion of household samples reflected the quality of the source itself. Despite the source being contaminated, most households in Area A had clean drinking-water, reflecting good management at the household level. As noted previously, the past incidence of diarrhoeal epidemic possibly had effect on handling/storage of household water in Parinche. However, polluted household samples from Area C, despite a clean source (Konkewadi with a past incident of diarrhoeal epidemic), indicate more focus on health education. No difference was found in the quality of water from families belonging to scheduled tribes and other families in both cases when provided water was clean or polluted.

Interventions at households are required for the constant availability of clean drinking-water and also to avoid recontamination (15,16). Thus, some simple interventions were suggested. These included the use of a one-inch coil of copper wire with a surface area of 8 cm^2^ for at least eight hours, use of mother solution, fitting of tap to storage vessel, and use of ladle to draw water. Clasen *et al.* point that the interventions to improve the quality of water not only depend on its effectiveness but also on other factors, such as affordability, acceptabili-ty, and sustainability (16). Hence, the preference of the community for copper wire (Table 5) can be attributed to its antibacterial (also reflected in our data) and oligodynamic action (17-20), its affordability, traditional usage, and relative zero-maintenance and effort involved. Moreover, the ability of copper to effect efficient disinfection without compromising the taste of water lent it a distinct advantage over mother solution whose use was limi-ted, particularly in isolated villages in Area C, due to the unavailability of bleaching powder or lack of time and effort to prepare the solution. Fixing a tap, however, was also a costly alternative (approximately 75 Indian Rupees/fitting) for the people in Area C. This argues for the devising of integrated and precise health education beyond a single intervention. By the end of the study period, it was noted that several households began storing water at a higher level. This package of interventions differed from other reported interventions, such as filtration, hand-washing with soap, solar disinfection, and flocculation-disinfection, use of devices, such as ceramic candle or iodine resin gravity filter (21-27).

There was no significant seasonal difference in the quality of water. However, despite drawing water from a clean source, contamination of household supplies was observed largely in the summer months due to repeated handling of water stocks—a cue for health messages to be more visible and intense in presummer months.

The relationship between the prevalence of diarrhoea and the quality of drinking-water is more complex. Most water treatment/storage interventions, sanitation practices, and health education in several other studies have been shown to be effective, and yet high-indicator bacteria counts were seldom associated with diarrhoea (28,29). In addition, as people can become infected with diarrhoeal organisms in multiple ways, transmission of diarrhoea may not be intercepted by improving the quality of water alone (16). Paradoxically, an increase in the annual diarrhoeal prevalence from 4.6% to 9.1% was observed at the end of the study period (data not shown). The extent of diarrhoeal diseases in the population, therefore, did not coincide with the efforts for the improved quality of water, indicating that the conventional potability indicators may not be truly reflective. Other factors, such as the immune status of the community and interplay of bacterial species in the water sources, may also play an important role. These issues, along with approaches towards improved sanitation and handling practices at the household level, could possibly impact the level of diarrhoeal diseases in the community.

Although this study describes a facilitating approach towards accessing potable drinking-water by rural communities, some limitations need to be mentioned. These include the sustainability of the approach and activities in the absence of an active implementing agency. The small sample size precluded stratification of communities and households for a more incisive analysis of community perspectives. Moreover, due to technical reasons, such as no water supply at time of samp-ling, electricity failure, tank valve damaged/under repair, etc., it was not always possible to get concurrent observations at source, collection point, and household levels. The long distance between the collection point and the testing site often affected the quality of samples through prolonged delays. This highlights the need to foster a certain level of technical capacity within a community or at least in its proximity.

**BOX**

- A petition was filed by the people of Parinche, New Hargude, and Kambalwadi (Area A and B) with the village developing officer after receiving information that water from the nearby Veer dam was being provided to the town of Saswad 30 km away without channelization to the local drought-affected villages. The issue was also covered by the local newspapers.
- In New Hargude (Area B), the construction of a tank was undertaken by a local contractor, resulting in improved workmanship with reduction of cost and better long-term maintenance.
- Due to a tank valve in Kambalwadi (Area B) being damaged, water could not be retained for chlorination. On realization of the importance of chlorination by the community, a new valve was sanctioned at the village meeting and replaced with the help of the FRCH staff.
- In the same village, a farmer sacrificed his crop and reserved well-water as a source of drinking-water for the village.
- In Parinche (Area A), repair to a damaged handpump was delayed, as no spare cylinder was available at the *Gram Panchayat.* People then voluntarily contributed US$ 22 per family and replaced the new cylinder.
- People in Khengarewadi (Area B) objected to the provision of water from a nearby minor irrigation tank to a receiving station instead of their village. On the refusal of the contractor to stop work, they discussed the issue in the village and decided to fight for their rights in the local village meetings (*Gram Panchayat*). Although men were initially hesitant, women in the village contacted the concerned officer and questioned him. Although they eventually failed to divert the supply, the issue increased the awareness of the entire community for their rights to adequate water.
- Eco-group children in Area C arranged for a proper drainage system from the tank that served to water the plants nearby, thus setting an example of water recycling.
- Desilting of the well in Area B was voluntarily done by women and school children, thereby inspiring the local youth to continue the activity.

Nevertheless, the interventions described here suggest that, during design of interventions and health messages, it is important to differentiate practices that are based on traditions from those that arise due to infrastructural inadequacy, poverty, and ignorance. The cyclical approach of multi-level surveillance, incorporating a two-way partnership with feedback to the community and imparting of technological competence to the community, was able to spark interest, capacity, and response in different segments of the community. This approach potentially can be scaled up to intermediate technical, social and institutional structures without the hazard from wasteful and inappropriate external inputs (1,30).
